# CircRNA_0001795 sponges miRNA-339-5p to regulate yes-associated protein 1 expression and attenuate osteoporosis progression

**DOI:** 10.1080/21655979.2021.2022074

**Published:** 2022-01-18

**Authors:** Mingyi Li, Chenxia Li, Huarong Zheng, Zhen Zhou, Wenjian Yang, Yu Gong, Xia Wu, Leyu Li

**Affiliations:** a Department of Endocrine, Xiangyang No. 1 People’s Hospital, Hubei University of Medicine, Xiangyang, China; b Department of Orthopaedics, Xiangyang No. 1 People’s Hospital, Hubei University of Medicine, Xiangyang, China

**Keywords:** Osteoporosis, circ_0001795, miR-339-5p, YAP1, osteogenic differentiation

## Abstract

Osteoporosis (OP) is one of the most common bone diseases, especially in women after menopause. Increasing evidence shows that non-coding RNAs are implicated in the pathogenesis of OP. In this study, based on the published circular RNA profiling data between OP patients and healthy controls, we found that circRNA_0001795 (circ_0001795) is downregulated in OP samples, which was further validated in the OP samples collected in this study. We therefore investigated the functional role and molecular mechanism of circ_0001795 in the osteogenic differentiation of human bone marrow stromal cells (hBMSCs) hBMSCs by alkaline phosphatase (ALP) activity assay, ALP and Alizarin Red S (ALS) Staining, luciferase reporter assay. Our data revealed that the overexpression of circ_0001795 could significantly promote the osteogenic differentiation of hBMSCs. MiRNA-339-5p (miR-339-5p) was identified as a target of circ_0001795, and miR-339-5p mimic attenuated the effect of circ_0001795 overexpression. MiR-339-5p downregulated yes-associated protein 1 (YAP1), which mediates the effect of circ_0001795 overexpression. Overall, this study uncovered the role of circ_0001795/miR-339-5p/YAP1 axis in regulating osteogenic differentiation, indicating that targeting Circ_0001795 could serve as a novel therapeutic target for OP.

## 1. Introduction

Osteoporosis (OP) is the most common bone disease with a gradual decrease of bone mass, which frequently occurs in women after menopause [1,2]. The main cause of OP is the abnormal activation of osteoclasts which are responsible for bone resorption [3]. According to statistics, so far there are about 20 million people suffering from various degrees of OP [4,5]. The treatment of OP has benefited from advancement of medical therapies [6]. However, due to the complex pathogenesis of OP, understanding the molecular mechanisms underlying OP progression will provide insights into the novel intervention strategies for ameliorating OP progression [3,7].

CircRNAs are a type of non-coding RNA, which are widely expressed in various human tissues and participate in the regulation of the pathogenesis and progression of various diseases [8,9]. CircRNAs are implicated in the regulation of cell proliferation, migration, invasion, apoptosis, and other biological activities [10,11]. CircRNAs can function as molecular sponge for microRNAs (miRNAs) by competitively binding with miRNA and interfering its interaction with messenger RNAs (mRNAs), thereby regulating the expression of target mRNAs involved in different cellular responses [12–14]. Previous studies have identified several circRNAs and investigated their functions in bone diseases and cancers. For instance, circRNA_33186 is highly expressed in osteoarthritis (OA) and can promote the progression of OA [15]. CircMTO1 has been found to inhibit the migration and infiltration of liver cancer cells by acting as a molecular sponge of miRNA-9 [16]. CircRNA SLC8A1 plays an inhibitory role in both bladder cancer and cardiac hypertrophy [17,18]. There are also a few reports regarding the roles of circRNA dysregulation in OP. CircRNA_0016624 can regulate and enhance the expression of BMP2 through miRNA-98 in OP, suggesting its potential to prevent OP [19]. In addition, circRNA_0048211 was reported to reduce the process of postmenopausal osteoporosis (PMOP) by modulating BMP2 expression through miRNA-93-5p [20]. In addition, there is a report showing that circRNA_0001795 (circ_0001795) is significantly downregulated in OP patients [21].

MiRNAs are another class of small non-coding RNAs [22], which may function downstream of circRNAs. In a study of osteosarcoma, it was found that miRNA-339-5p (miR-339-5p) can mediate the regulation of TGF-β1 expression by lncRNA NEAT1 [23]. The downregulation of miR-339 was reported to promote the osteogenic differentiation of BMSCs by targeting DLX5, thereby alleviating OP [24]. However, whether miR-339 also targets other downstream mRNAs in OP remains to be investigated.

Yes-associated protein 1 (YAP1) is a transcription factor and negatively regulated by Hippo pathway, which is involved in osteodegenerative diseases, such as OP and OA [25]. The expression of YAP1 seems to be regulated by circRNAs and miRNAs. A previous study showed that circ-ITCH upregulates YAP1 by acting as a molecular sponge of miRNA-214 to promote osteogenesis [26]. In addition, circRNA_0024097 can promote osteogenesis through miRNA-376b-3p/YAP1 axis and Wnt pathway, thereby ameliorating OP [27]. Therefore, YAP1 seems to be an important transcription factor under complex regulation in the pathological process of OP.

Based on the analysis of the published circular RNA profiling data between OP patients and healthy controls by a previous study (GSE161361), we found that circ_0001795 was downregulated OP patients, which was further validated by the clinical samples collected in our study. We hypothesized that the reduced expression of circ_0001795 is implicated in the progression of osteoporosis by regulating osteogenic differentiation. Based on human bone marrow stromal cells (hBMSCs) differentiation model, our data demonstrated that circ_0001795 overexpression promoted the osteogenic differentiation of hBMSCs. MiR-339-5p was identified as a target of circ_0001795 to mediate the osteogenic effect of circ_0001795. MiR-339-5p downregulated yes-associated protein 1 (YAP1), which mediates the osteogenesis induced by circ_0001795 overexpression. Collectively, our study revealed the role of circ_0001795/miR-339-5p/ YAP1 axis in osteogenic differentiation, indicating that targeting Circ_0001795 could serve as a novel therapeutic target for OP.

## 2. Materials and methods

### 2.1. Clinical sample collection

The bone marrow tissue samples were collected from 30 OP patients who were diagnosed as osteoporosis in Xiangyang No. 1 People’s Hospital and 20 age-matched healthy controls. All participants did not have chronic diseases such as V_D_ deprivation and diabetes or did not receive long-term medication. Osteoporosis was diagnosed using the World Health Organization parameters by examining the biopsy specimen of bone marrow tissue from bones of the pelvis. All the bone marrow samples were collected and stored in liquid nitrogen. Informed consent was obtained from all participants. The clinical procedure and sample collection were approved by the ethics committee of Xiangyang No. 1 People’s Hospital (2020LC098).

### 2.2. Cell culture and transfection

The human bone marrow mesenchymal stem cells (hBMSCs) used in this study were cultured at 37°C and 5% CO_2_. Cells were cultured with α-MEM medium (Gibco, USA), supplemented with 2 mM L-glutamine, 10% fetal bovine serum (FBS; Gibco, USA), and 1% penicillin and streptomycin (Gibco, USA). The osteogenic differentiation induction medium was the cell culture medium containing 200 μM ascorbic acid, 10 mM β-glycerophosphate, and 100 nM dexamethasone (all from Sigma, St Louis, MO, USA). Cells were harvested and analyzed at 0, 7th, 14th, 21th days for circ_0001795 expression analysis.

In order to knockdown YAP1 and miR-339-5p, specific siRNA, and microRNA inhibitor were used: si-YAP1 (5ʹ-GGCCCUUUGAUUUAGU AUA-3ʹ), miR-339-5p inhibitor (5ʹ-CGUGAGCUCCUGGAGGACAGGGA-3ʹ), negative control siRNA (si-NC): 5ʹ-CGUACGCGGAAUACUUCGA-3ʹ, negative control for inhibitor (inhibitor-NC): 5ʹ-CAGUACUUUUGUGUAGUACAA-3ʹ) were synthesized by GenePharma (Shanghai, China). In order to overexpress circ_0001795, pcDNA 3.1 circRNA mini vector was constructed. MiR-339-5p mimic (5ʹ-UCCCUGUCCUCCAGGAGCUCACG-3ʹ) and its negative control (mimics-NC: 5ʹ-ACGUGACACGUUCGUAGAATT-3ʹ) were purchased from GenePharma.

Cell transfection was performed using Lipofectamine® 3000 reagent (Thermo Fisher Scientific, L3000001). Briefly, in 6 well plate, 70% confluent cells were transfected with 100 nM of microRNA mimic/inhibitor, siRNA or 6 ug of pcDNA3.1-lncRNA SNHG16 plasmid according to manufacturer’s instruction. Transfected cells were subjected to subsequent analysis 48 hours post-transfection.

### 2.3. RNA extraction and RT-qPCR

The total RNA was extracted using TRIzol (Invitrogen, 15,596,026). The extracted total RNA was dissolved in DEPC water and its concentration was measured with NanoDorp. 5 μg of total RNA was used for reverse-transcription by HiScript III 1st Strand cDNA Synthesis Kit (Vazyme, China). qPCR was carried out using AceQ qPCR SYBR Green Master Mix (Vazyme, China) in a 7500 Real-Time PCR System (Applied Biosystems, Carlsbad, CA, USA). The relative expression was calculated by the 2-ΔΔCt method, using GAPDH as an internal reference. The primers used in this study were synthesized by Shanghai Shenggong Biological Engineering Co., Ltd as follows: circ_0001795: 5ʹ-GTGTCCGTGGATGCGGAGG-3ʹ (forward) and 5ʹ-CTGCTGCTCCCGTGAGCGG-3ʹ (reverse); miR-339-5p: 5ʹ-GTGTCCCTGTCCTCCAGG-3ʹ (forward) and 5ʹ-GTGCAGGGTCCGAGGT-3ʹ (reverse); YAP1: 5ʹ-TTCGGCAGGCAATACGGAAT-3ʹ (forward) and 5ʹ-GTTGAGGAAGTCGTCTGGGG-3ʹ (reverse); GAPDH: 5ʹ-CAAGGTCATCCATGACTTTG-3ʹ (forward) and 5ʹ-GTCCACCACCCTGTTGCTGTAG-3ʹ (reverse).

### 2.4. Nuclear/cytoplasmic fractionation

Cytoplasmic & Nuclear RNA Purification Kit (Amyjet scientific) was used to separate the cytoplasmic and nuclear RNA in hBMSCs according to the user manual as previously described [15]. Subsequently, qRT-PCR was used to detect relative abundance of circ_0001795 in the cytoplasmic and nuclear fraction, with GAPDH and U6 gene as internal controls, respectively.

### 2.5. Western blot assay

Cells were lysed with pre-cooled RIPA lysis buffer (Beyotime biotechnology, China) as previously described [15]. Lysed cells were centrifuged at 14,000 rpm for 10 mins. The supernatant containing total protein lysate was quantified by a BCA Protein assay kit (Beyotime Biotechnology P0009; Shanghai, China). 10 µg protein was used for SDS-PAGE electrophoresis and transferred to PVDF membrane. The membrane was blocked with 5% skimmed milk for 1 h. Then the membranes were incubated with primary antibodies YAP1 (dilution ratio 1:1000), Runx2 (dilution ratio 1:1000), OCN (dilution ratio 1:1000), OPN (dilution ratio 1:1000) and GAPDH (dilution ratio 1:5000) (All from Santa cruz, USA) overnight at 4°C. After washes with TBST buffer, the membranes were further incubated with secondary antibody labeled with horseradish peroxidase (HRP) (1:3000; Cell signaling #7074, MA, USA) for 1 h at room temperature. Finally, ECL luminescent solution (Santa Cruz, TX, USA, sc-2048) was used for band development and the membrane was imaged using Gel-Doc 200 system (Bio-Rad, CA, USA).

### 2.6. Dual luciferase reporter assay

The wild-type (WT) or mutant (Mut) circ_0001795 sequence, and WT or Mut 3ʹUTR of YAP1 containing the predicted binding site of miR-339-5p were cloned into the pmirGLO luciferase reporter vector, respectively (Promega, Madison, USA). The reporter plasmid and Renilla luciferase (hRlucneo) control plasmid were co-transfected into cells with miR-NC or miR-339-5p mimic in a 12-well plate (1 × 10^5 cells/well) using Lipofectamine 3000 reagent (Invitrogen, L3000001). 48 h post transfection, the relative luciferase activities were measured using Dual-Luciferase Reporter Assay Kit (Promega, E1910) on a luminescence microplate reader (Infinite 200 PRO; Tecan). The relative firefly luciferase activity in the reporter plasmid was normalized to that of Renilla luciferase (hRlucneo) control plasmid.

### 2.7. RNA pull-down assay

Biotinylated circ_0001795 probe or control probe were used for RNA pull-down assay. 1 × 10^6^ Cells were lysed in RIPA lysis buffer on ice for 15 mins. Then, 50 µL of M-280 streptavidin magnetic beads (Sigma-Aldrich, 11205D) were incubated with 0.5 µg of biotinylated circ_0001795 or control probe for 30 min in the lysis buffer. Subsequently, the above mixture was incubated with the cell lysate at 4°C for 4 h. 10% of total cell lysates was saved as the input. A magnetic bar was used to pull down the magnetic beads and associated nucleic acids, then the samples were washed 4 times with high salt wash buffer. Both the input and the elutes from the pull-down were purified with Trizol reagent (Invitrogen, 15,596,026) according to the manufacturer’s protocol. The relative level of precipitated miR-339-5p was quantified by RT-qPCR.

### 2.8 Fluorescence in situ hybridization (FISH)

In situ hybridization was performed using specific probes for circ_0001795. 5ʹCY3-labeled circ_0001795 probe crossed the junction site of circ_0001795 were designed and synthesized by Shanghai Shenggong Biological Engineering Co., Ltd. Hybridization assay was performed by FISH Kit (Genepharma, China) according to the manufacturer’s protocol. hBMSCs were fixed with 4% paraformaldehyde and permeabilized with 0.5% Triton-X100. Fixed cells were incubated with 100 nM probes at 37°C for 12 h. The nuclei were counterstained by DAPI. Signals were detected by an inverted fluorescence microscope (OLYMPUS, Japan).

### 2.9 DNA sequencing

RNA was reverse-transcribed into cDNA using HiScript III 1st Strand cDNA Synthesis Kit (Vazyme, China). Polymerase chain reaction (PCR) was performed to amplify the target sequence using 2× Taq Master Mix (Vazyme, China) according to the manufacturer’s protocol. PCR products were sequenced by DNA sequencer (ABI3500, USA).

### 2.10. Alkaline phosphatase (ALP) activity assay

ALP activity was evaluated by colorimetric assay as previously described [26]. The hBMSCs were differentiated for 3 days and 6 days under the specified conditions. 1 million cells were collected and lysed in 200 µL RIPAbuffer, and subsequently centrifuged at 10,000 × g for 15 min at 4°C. Then, 5 μl of supernatant was mixed with 5 μl of ALP substrate (BD Biosciences Clontech, USA) and 15 μl of LUPO buffer. The mixture was incubated for 40 min at room temperature, and then the ALP activity was determined on a Synergy H1 microplate reader (Winooski, Vermont, USA).

### 2.11. ALP staining assay

ALP staining assay was evaluated as previously described [26]. HBMSCs were seeded in 24-well plates at the density of 2 × 10^5^ cell/well under 7 days’ osteogenic induction. The cells were washed with PBS, fixed with 4% paraformaldehyde and permeabilized with 0.5% Triton-X100. Fixed cells were incubated with a basic dye mixture (Leagene Biotech., Beijing, China) in the dark for 40 min at room temperature. Finally, images were captured by an inverted fluorescence microscope (OLYMPUS, Japan).

### 2.12. Alizarin red staining (ARS) assay

ARS assay is evaluated as previously described [26]. HBMSCs were seeded in 24-well plate at the density of 2 × 105 cell/well under 15 days’ osteogenic induction. The cells were then stained with ARS dye (Leagene Biotech., Beijing, China) for 10 min at room temperature, and images were captured by an inverted fluorescence microscope (OLYMPUS, Japan). For quantitative analysis, ARS was extracted with 10% acetic acid (Sigma, USA) and measured at 405 nm on a Synergy H1 microplate reader (Winooski, Vermont, USA).

### 2.13. Data analysis

Data analysis was carried out using GraphPad Prism 6.0 software. The statistical difference between two groups was compared using unpaired student’s t tests. Comparisons among multiple groups were analyzed using one-way analysis of variance (ANOVA) with Tukey’s post hoc test for pairwise comparison. The correlation of expression was statistically analyzed by Spearman correlation coefficient analysis. All experimental data are presented as the mean ± SD of at least three independent experiments. P < 0.05 was considered statistically significant. */^p < 0.05, **/^^p < 0.01, ***/^^^p < 0.001.

## 3. Results

In this study, we hypothesized that the reduced expression of circ_0001795 is implicated in the progression of osteoporosis by regulating osteogenic differentiation. Based on the osteogenic differentiation model of hBMSCs, we demonstrated that circ_0001795 overexpression enhanced the osteogenic differentiation of hBMSCs and identified miR-339-5p a downstream target of circ_0001795. MiR-339-5p downregulated yes-associated protein 1 (YAP1) to mediate the osteogenic effect of circ_0001795 overexpression. Our data highlighted the role of circ_0001795/ miR-339-5p/ YAP1 axis in osteogenic differentiation.

### 3.1. Circ_0001795 is downregulated in bone marrow samples of OP patients

**Figure 1.**
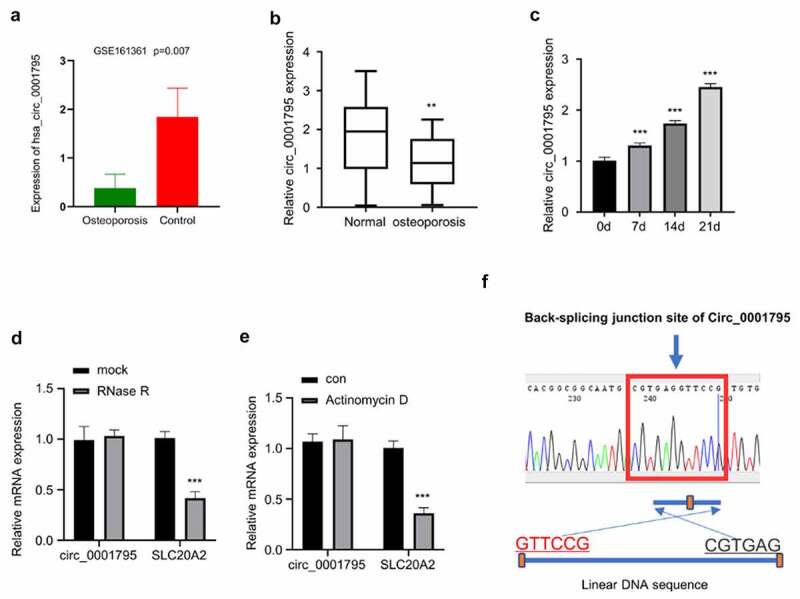
Circ_0001795 is downregulated in OP patients and is upregulated during osteogenic differentiation. (a) Based on the analysis of circRNA profiling of exosomes isolated from OP patients (N = 3) and healthy controls (N = 3) (GSE161361), circ_0001795 was significantly downregulated in OP patients. (b) Circ_0001795 expression was analyzed in bone marrow tissue samples of 30 OP patients and 20 healthy controls by qRT‐PCR. (c) Circ_0001795 expression was analyzed by qRT‐PCR during osteogenic differentiation for 0, 7th, 14th, 21th days. (d) Circ_0001795, rather than linear SLC20A2, resisted to Rnase R digestion in hBMSCs. (e) Relative expression of circ_0001795 and linear SLC20A2 in hBMSCs treated with actinomycin D at 24 h. (f) Result of sanger sequence showed the back-splicing pattern and junction site of circ_0001795.

Based on the analysis of circRNA profiling of exosomes isolated from OP patients (N = 3) and healthy controls (N = 3) (GSE161361), we found that circ_0001795 was significantly downregulated in OP patients (Figure 1(a)) In order to confirm the downregulation of circ_0001795 in OP patients, we collected 30 bone marrow samples from 30 OP patients and 20 samples from age-matched healthy controls. The RT-qPCR results showed that circ_0001795 was significantly downregulated in OP patients as compared with healthy controls (Figure 1(b)). The patients were divided into circ_0001795 high expression (n = 15) and low expression (n = 15) group based on the median level of circ_0001795 expression. The clinical parameters of the two groups were summarized in Table 1. In the low expression group, the T-Score Lumbar Spine (L1-L4) was significantly lower than the high expression group. To demonstrate the implication of circ_0001795 in osteogenesis, hBMSCs were cultured under osteogenic conditions for different periods, and RT-qPCR analysis revealed that the expression of circ_0001795 gradually increased with the osteogenic differentiation time (Figure 1(c)). In order to validate the existence of circ_0001795, we used qRT-PCR to quantify the level of circ_0001795 and the SLC20A2 mRNA (the gene locus for circ_0001795) after RNase R treatment. Circ_0001795 was resistant to RNase R digestion while SLC20A2 mRNA was significantly reduced by RNase R (Figure 1(d)). Similarly, SLC20A2 mRNA expression decreased after Actinomycin D treatment, but circ_0001795 did not change significantly (Figure 1(e)). These data suggest that circ_0001795 has a circular form and is not transcribed by RNA polymerase II. The circular format of circ_0001795 was further confirmed by Sanger sequencing, which showed the junction site of Circ_0001795 as compared to the linear SLC20A2 sequence (Figure 1(f)).

**Table 1.** Characteristics of osteoporosis clinical samples

Clinical index (mean ± SD)	Circ_0001795 low expression (n = 15)	Circ_0001795 high expression (n = 15)
Age	60.2 ± 7.32	61.4 ± 7.40
Height (cm)	158 ± 2.95	154 ± 3.64
Weight (kg)	58 ± 3.12	60.2 ± 2.23
BMD (g/cm2)	0.78 ± 0.06	0.84 ± 0.25
T-Score Lumbar Spine (L1-L4)	−2.5 ± 0.85	−1.2 ± 0.75**

**Difference is significant at the 0.01 level (2-tailed).

### 3.2. Overexpression of circ_0001795 promotes osteogenic differentiation of hBMSCs

**Figure 2.**
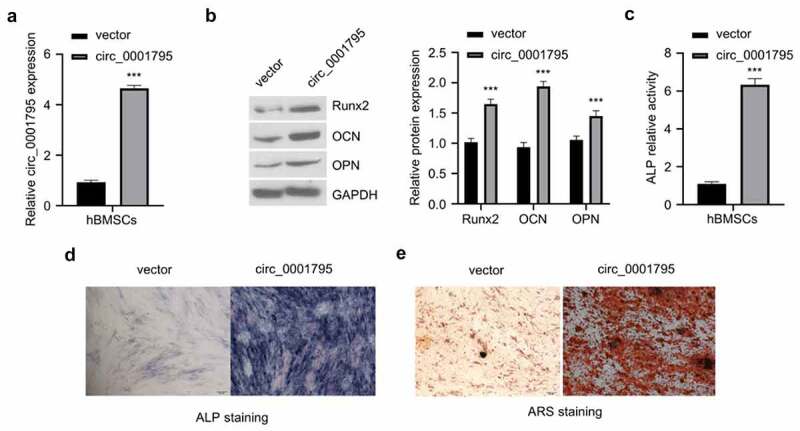
Overexpression of circ_0001795 promotes osteogenic differentiation of hBMSCs. (a) Overexpression efficacy of circ_0001795 expression vector was examined in hBMSCs by qRT‐PCR. (b) The protein levels of Runx2, OCN and OPN were analyzed by Western blot upon the overexpression of circ_0001795. (c-e) Osteogenic differentiation of hBMSCs was evaluated by ALP activity, ALP staining and ARS staining upon the overexpression of circ_0001795.

In order to investigate the functional role of circ_0001795 in OP, we constructed an overexpression plasmid of circ_0001795 and verified its overexpression effect after transfection (Figure 2(a)). The overexpression of circ_0001795 significantly increased the protein levels of osteogenic markers such as Runx2, osteocalcin (OCN), and osteopontin (OPN) in hBMSCs cells (Figure 2(b)). Functionally, circ_0001795 overexpression also significantly increased ALP activity (Figure 2(c)). The results of ALP staining showed consistent results (Figure 2(d)). To quantify the mineralization by osteogenic differentiation, we performed ARS staining and found that the overexpression of circ_0001795 significantly also dramatically augmented the mineralization of osteogenic differentiation (Figure 2(e)). The above results indicate that overexpression of circ_0001795 promotes the osteogenic differentiation of hBMSCs.

### 3.3. Circ_0001795 targets miR-339-5p in osteogenesis

**Figure 3.**
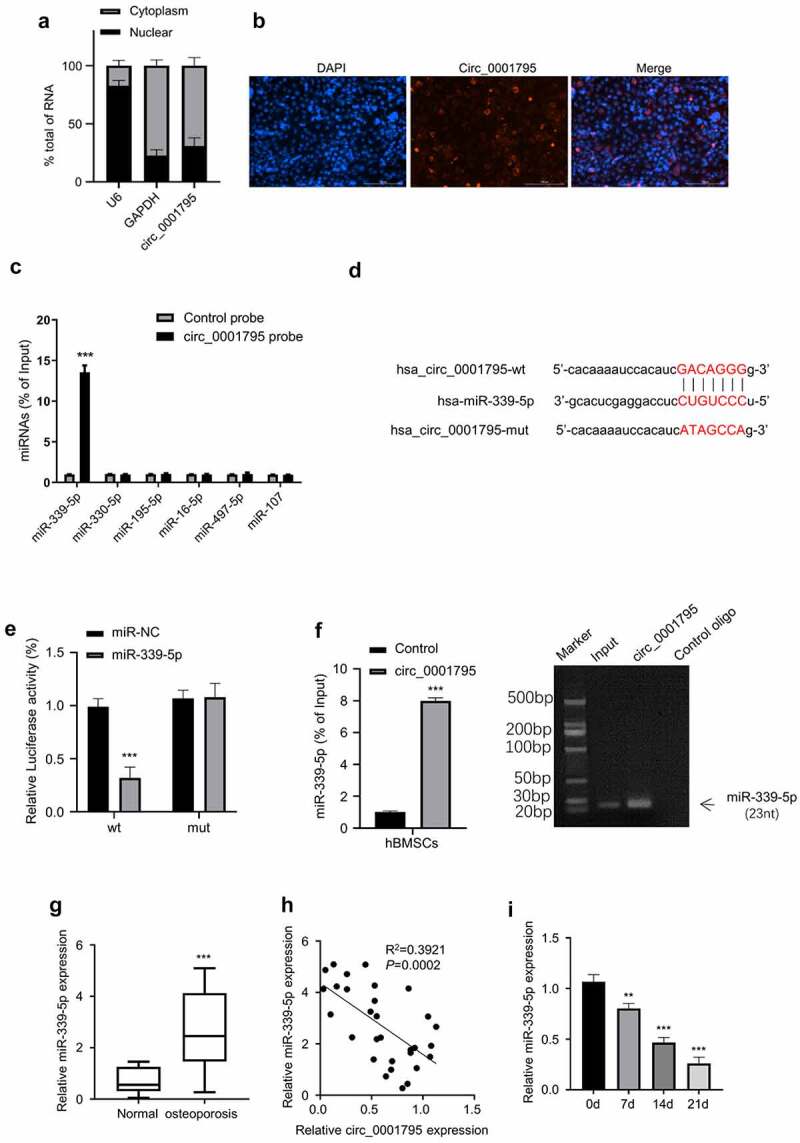
Circ_0001795 targets miR-339-5p. (a) The percentage of circ_0001795, GAPDH and U6 in the cytoplasmic and nuclear fractions were analyzed in hBMSCs by qRT-PCR. (b) Localization of circ_0001795 was determined in hBMSCs by FISH, nuclei were stained with DAPI, scale bar, 200 μM. (c) Biotinylated circ_0001795 probe could capture miR-339-5p among six miRNA candidates by RNA pull-down analysis. (d) StarBase analysis showed the potential binding sites between circ_0001795 and miR-339-5p. (e) and (f) The interaction between circ_0001795 and miR-339-5p was validated by luciferase reporter assay and RNA pull-down in hBMSCs. (g) The expression of miR-339-5p was analyzed in bone marrow tissue samples of 30 OP patients and 20 healthy controls by qRT‐PCR. (h) The correlation of circ_0001795 and miR-339-5p expression level was analyzed in bone marrow tissue samples of 30 OP patients. (i) The expression of miR-339-5p was analyzed by qRT‐PCR during the time course of osteogenic differentiation on 0, 7, 14 and 21 day.

The localization of circRNA in the cell indicates a different mechanism of actions. We next determined the relative abundance of circ_0001795 in the nuclear and cytoplasmic fractions. Circ_0001795 was predominantly localized in the cytoplasm (Figure 3(a)), which was confirmed by RNA FISH staining (Figure 3(b)). To search for its potential interacting miRNAs, we used the Starbase database to predict that the miRNAs that circ_0001795 might target. Among the six miRNAs candidates, circ_0001795 probe was able to significantly enrich miR-339-5p (Figure 3(c,d)). To further confirm this interaction, we performed dual-luciferase activity assay using WT and mutated circ_0001795 reporter. The co-transfection of miR-339-5p inhibited the luciferase activity of circ_0001795-WT reporter, but no inhibition was observed for circ_0001795-Mut reporter (Figure 3(e)). We confirmed that circ_0001795 probe could pull-down miR-339-5p by both RT-qPCR and agarose gel electrophoresis (Figure 3(f)). In addition, we found that miR-339-5p was highly expressed in the samples of OP patients as compared with that in the healthy controls (Figure 3(g)). Spearman correlation coefficient analysis showed that in 30 cases of OP, there was a significant negative correlation between the expression levels of circ_0001795 and miR-339-5p (Figure 3(h)). The expression level of miR-339-5p gradually decreased during the process of osteogenic differentiation (Figure 3(i)). Together, these data suggest that Circ_0001795 targets miR-339-5p in osteogenesis.

### 3.4. Circ_0001795 promotes osteogenic differentiation of hBMSCs by regulating the expression of miR-339-5p

**Figure 4.**
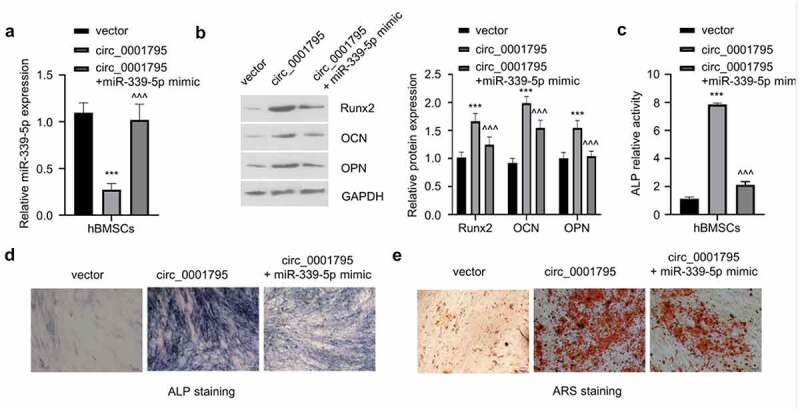
Circ_0001795 promotes osteogenic differentiation of hBMSCs by regulating the expression of miR-339-5p. (a) The relative expression of miR-339-5p was analyzed in hBMSCs upon the overexpression of circ_0001795, in the presence or absence of miR-339-5p mimic. (b) The expression of Runx2, OCN and OPN were analyzed in hBMSCs following circ_0001795 overexpression, in the presence or absence of miR-339-5p mimic. (c-e) Osteogenic differentiation of hBMSCs was evaluated by ALP activity, ALP staining and ARS staining upon overexpression of circ_0001795, in the presence or absence of miR-339-5p mimic.

To confirm the involvement of miR-339-5p in circ_0001795 mediate osteogenesis, we transfected miR-339-5p mimic in circ_0001795 overexpressing cells. The overexpression of circ_0001795 significantly reduced the expression of miR-339-5p, while the co-transfection of miR-339-5p could restore the level of miR-339-5p (Figure 4(a)). The co-transfection of miR-339-5p mimic could impair the increase of Runx2, OCN, and OPN induced circ_0001795 overexpression (Figure 4(b)). Similarly, overexpression of miR-339-5p also significantly attenuated the increase in ALP activity caused by the overexpression of circ_0001795 (Figure 4(c,d)), as well as the level of ARS staining (Figure 4(e)). These results clearly indicate that miR-339-5p mediates the osteogenic differentiation of hBMSCs under circ_0001795 overexpression.

### 3.5. MiR-339-5p negatively regulates YAP1 expression

**Figure 5.**
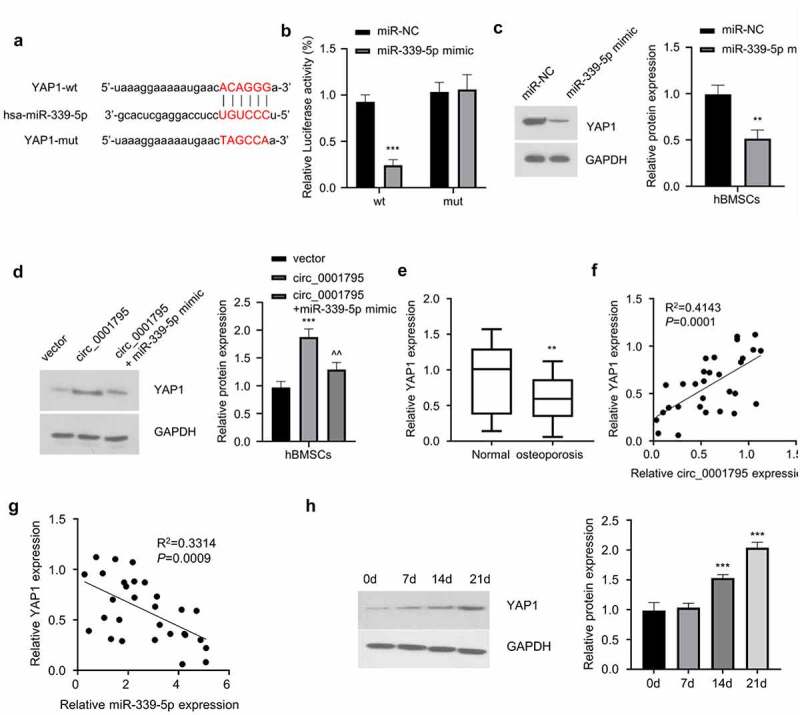
MiR-339-5p targets YAP1. (a) StarBase analysis showed the potential binding site for miR-339-5p and 3ʹUTR of YAP1 mRNA. (b) The interaction between miR-339-5p and 3ʹUTR of YAP1 mRNA was validated by luciferase reporter assay in hBMSCs in the presence of absence of miR-339-5p mimic. (c) The effect of miR-339-5p on expression of YAP1 was analyzed in hBMSCs upon the overexpression miR-339-5p. (d) The expression of YAP1 was analyzed by Western blot in hBMSCs following circ_0001795, in the presence or absence of miR-339-5p mimic. (e) The relative expression of YAP1 was analyzed in bone marrow tissue samples of 30 OP patients and 20 healthy controls by qRT‐PCR. (f) The correlation of circ_0001795 and YAP1 expression level was analyzed in bone marrow tissue samples of 30 OP patients. (g) The correlation of miR-339-5p and YAP1 expression level was analyzed in bone marrow tissue samples of 30 OP patients. (h) The expression of YAP1 was analyzed by Western blot during the time course of osteogenic differentiation on 0, 7, 14 and 21 day.

To further identify the molecular target of miR-339-5p, we first identified that miR-339-5p could potentially bind to the 3ʹUTR of YAP1 mRNA through the Starbase database (Figure 5(a)). To further confirm the interaction between miR-339-5p and 3ʹUTR of YAP1, we performed dual-luciferase reporter assay and found that as compared with miR-NC, overexpression of miR-339-5p could inhibit luciferase activity of YAP1-WT reporter, while no effect was observed when the predicted binding site in the 3ʹUTR of YAP1 was mutated (Figure 5(b)). Consistently, the overexpression of miR-339-5p significantly reduced expression of YAP1 (Figure 5(c)). In the meanwhile, circ_0001795 overexpression could increase the protein level of YAP1, while miR-339-5p mimic partially reduced YAP1 level (Figure 5(d)). In the clinical samples, YAP1 expression was also significantly lower in OP patients (Figure 5(e)), and Spearman correlation coefficient analysis showed that YAP1 expression level was positively correlated with the expression of circ_0001795 (Figure 5(f)), while it was negatively correlated with the expression of miR-339-5p (Figure 5(g)). During the process of osteogenic differentiation, the expression of YAP1 gradually increased with time (Figure 5(h)). Together, these data indicate that circ_0001795/miR-339-5p regulates YAP1 expression during osteogenic differentiation.

3.6 Knockdown of YAP1 partially reverses the effect of miR-339-5p inhibitor on the osteogenic differentiation of hBMSCs

**Figure 6.**
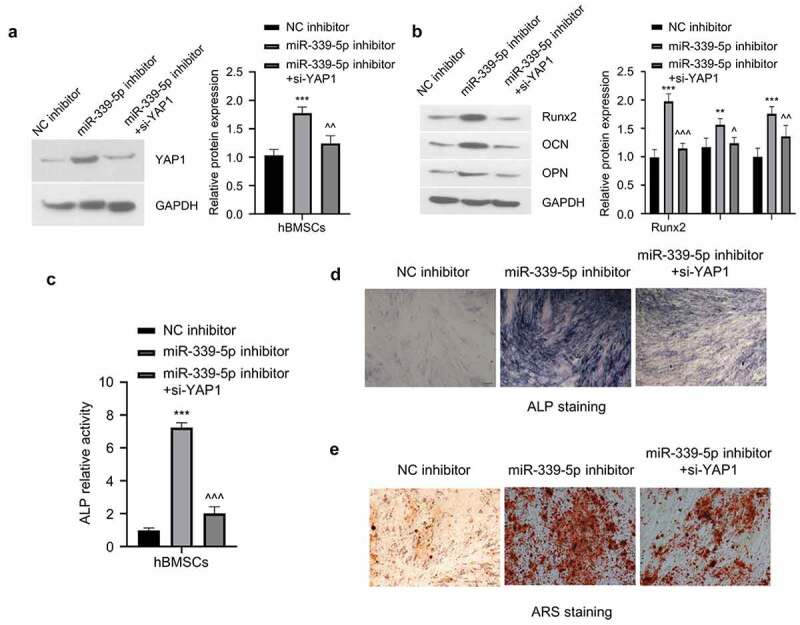
Knockdown of YAP1 partially reverses the effect of miR-339-5p inhibitor on the osteogenic differentiation of hBMSCs. (a) The expression of YAP1 in hBMSCs following treatment of miR-339-5p inhibitor with or without si-YAP1 silencing. (b) The expression of Runx2, OCN and OPN were analyzed in hBMSCs following treatment of miR-339-5p inhibitor with or without si-YAP1 silencing. (c-e) Osteogenic differentiation of hBMSCs was evaluated by ALP activity, ALP staining and ARS staining upon treatment of miR-339-5p inhibitor with or without si-YAP1 silencing.

To functionally validate the involvement of YAP1 in osteogenic differentiation, we co-transfected si-YAP1 with miR-339-5p inhibitor. MiR-339-5p inhibitor increased YAP1 level which was partially reduced by YAP1 knockdown (Figure 6(a)). MiR-339-5p inhibitor could significantly increase the protein expression of Runx2, OCN, and OPN, and the co-transfection of si-YAP1 partially impaired the increase of Runx2, OCN, and OPN caused by miR-339-5p inhibitor (Figure 6(b)). Similarly, transfection of si-YAP1 also attenuated the increase in ALP activity caused by miR-339-5p inhibitor (Figure 6(c,d)) as well as the ARS staining (Figure 6(e)). These results altogether suggest that YAP1 is a downstream mediator of the osteogenic differentiation of hBMSCs under the regulation of miR-339-5p.

## 4. Discussion

OP is a chronic skeletal dysfunction, which is characterized by bone loss, microstructure destruction, and increased bone fragility in the elderly [28]. Due to the intensification of population aging in recent years, the incidence of fractures in the elderly associated with OP has increased. In recent years, the dysregulation of circRNAs are widely reported in a variety of human disease, which could serve as potential diagnosis biomarkers and treatment targets [8,9]. In particular, several circRNAs have been identified as regulatory molecules in the progression of OP. A previous study reported that circRNA_0006393 can activate the development of OP by regulating miR-145-5p, thereby affecting the expression of FOXO1 [29]. CircRNA_0016624 was found to regulate the expression of BMP2 by targeting miRNA-98, promoting its protective effect in OP [19]. Interestingly, a recent study indicated that circ_0001275 downregulation could be used as a diagnostic marker for OP, which is conducive to the clinical diagnosis of OP [30].

In this study, we investigated the role and regulation mechanism of circ_0001795 in osteogenesis. We first confirmed that circ_0001795 is downregulated in bone marrow samples from OP patients. Circ_0001795 is upregulated in osteogenesis model and its overexpression promotes osteogenesis. In addition, previous studies have shown that Alizarin red and ALP staining are often used to explore the osteogenic potential of hBMSCs. Our data revealed that overexpression of circ_0001795 can significantly promote the osteogenic differentiation of hBMSCs by Alizarin red and ALP staining. Therefore, we conclude that circ_0001795 promotes the osteogenic potential of hBMSCs. Combined with its downregulation in OP samples, our data indicate that circ_0001795 downregulation may be implicated in OP by impairing osteogenesis.

Based on the prediction of the downstream miRNAs of circ_0001795, we further identified miR-339-5p as a downstream regulatory molecule of circ_0001795 to play a role in osteogenesis. As many previous studies have shown, circRNA can affect the expression of its downstream target mRNA by sponging miRNAs, which are implicated in the progression of OP [19,29]. For example, miRNA-34a can alleviate OP and bone metastasis by inhibiting osteoclastogenesis and Tgif2 [31]. MiRNA-485-5p was found to activate OP in BMSCs by targeting Osterix [32]. MiRNA-542-3p can inhibit the progression of OP by reducing the expression of SFRP1 [33]. In this study, we validated the functional involvement of that miR-339-5p in osteogenesis by demonstrating its biding to circ_0001795, and circ_0001795 acts as a molecular sponge of miR-339-5p to regulate the ability miR-339-5p to negatively regulate YAP1 expression. YAP1 downregulation is widely involved in the occurrence and development of osteodegenerative diseases including OP [25]. Our data have also showed that YAP1 is significantly downregulated in patients with OP. Furthermore, the expression of YAP1 in hBMSCs is directly regulated by miR-339-5p and indirectly regulated by circ_0001795. The change of YAP1 expression modulates osteogenic differentiation of hBMSCs. Our data collectively pinpoint the role of circ_0001795/miR-339-5p/YAP1 axis in osteogenic differentiation. Future works need to focus on the validation of circ_0001795/miR-339-5p/YAP1 axis in osteogenic differentiation in the animal model of osteoporosis.

## 5. Conclusion

In conclusion, circ_0001795 acts as a molecular sponge of miR-339-5p to affect its regulation on YAP1 expression. Circ_0001795 is downregulated in clinical samples of OP patients and circ_0001795 overexpression promotes the osteogenic differentiation of hBMSCs. The changes of miR-339-5p or YAP1 expression also regulate osteogenic differentiation enhanced by Circ_0001795 overexpression. We conclude that circ_0001795 downregulation is implicated in the osteoporosis by regulating osteogenesis.

## Highlights

- circ_0001795 is downregulated in osteoporosis (OP) bone marrow samples.
- circ_0001795 overexpression enhances osteogenic differentiation.
- circ_0001795 sponges miR-339-5p to maintain YAP1 level and support osteogenesis
